# Lysosome trafficking across intracellular and intercellular networks in the brain

**DOI:** 10.3389/fnmol.2026.1755292

**Published:** 2026-02-02

**Authors:** Dylan T. Murphy, Chih Hung Lo

**Affiliations:** 1 Department of Biology, Syracuse University, Syracuse, NY, United States; 2 Interdiciplinary Neuroscience Program, Syracuse University, Syracuse, NY, United States

**Keywords:** cell-cell interaction, intrinsically disordered proteins (IDPs), lysosome acidification, lysosome trafficking, microtubule transport, neurodegenerative diseases, neuron-glia communication, tunneling nanotubes (TNTs)

## Introduction

While lysosomes have been traditionally viewed as degradative compartments, evidence suggests that they are signaling organelles that are highly mobile within or across cells to maintain local and global homeostasis (Settembre et al., 2013; Spencer et al., 2025). Spatial distribution of lysosomes has been shown to influence their acidification and functions (Ferguson, 2018a), which are essential to support normal cellular activities in the brain (Zeng et al., 2025; Asimakidou et al., 2024; Quick et al., 2023). Although lysosome transport is important in all neural cells, this is particularly critical in neurons, given their highly polarized and extended morphology. Lysosomal activity can vary across cellular compartments, such as the soma and axon, highlighting the need for organelle transport to meet compartment-specific functional demands (Ferguson, 2018b). To sustain this transport, neurons rely on regulated trafficking of lysosomes along microtubules and other cytoskeletal elements within the cells (Matteoni and Kreis, 1987). Beyond intracellular trafficking, intercellular transfer of organelles, such as through tunneling nanotubes (TNTs), is a unique mechanism where neurons, astrocytes, and microglia appear to be capable of transferring organelles including lysosomes to one another under stress conditions (Rostami et al., 2017; Scheiblich et al., 2024). Defective lysosomal acidification, function, and trafficking and associated accumulation of toxic intrinsically disordered proteins (IDPs) have emerged as a disease mechanism across neurodegenerative disorders, including Alzheimer's disease (AD) and related tauopathies and Parkinson's disease (PD; Lee et al., 2022; Lo and Zeng, 2023; Lo and Rubinsztein, 2024). Lysosomal trafficking defects indicate that neurodegeneration arises not only from organelle deacidification and impaired degradative function, but also from the failure to properly deliver lysosomes to regions of need within cells and from disrupted exchange of functional and damaged lysosomes between cells in the brain.

## Intracellular lysosome transport

In neurons, lysosome formation and positioning depend on long-range transport mediated by microtubule motors. Anterograde transport, the process of trafficking toward the axon termini, is driven by kinesins, specifically kinesin-1 and kinesin-3, which interact with lysosomal membranes through the BORC-ARL8-SKIP complex (Farías et al., 2017). Retrograde transport, moving in the direction toward the soma, is mediated by the GTPase Rab7, Rab-interacting lysosomal protein (RILP), and the dynein–dynactin complex as the motor (Jordens et al., 2001). These opposing motors continuously reposition lysosomes to maintain degradative homeostasis (Cabukusta and Neefjes, 2018; Farfel-Becker et al., 2019).

Spatial compartmentalization of lysosomes based on acidity adds another layer to the transport of these organelles within neurons. Axonal lysosomes are often in the process of maturation as they move closer to the soma, representing late endosomal intermediates (Ferguson, 2018a; Johnson et al., 2016). This maturation gradient works through pH modulation, where lysosomes moving from the distal axon start around a pH of 6 and increasingly acidify to around pH 4.5 as they move toward the soma (Ferguson, 2018a; Overly, 1996). Lysosomal acidification state can influence spatial distribution, as acidified lysosomes undergo retrograde transport toward the soma, with fully acidified lysosomes localizing near the nucleus. Less acidic lysosomes are localized in distal neuronal compartments and toward the periphery of the cell (Malik et al., 2019). Acidity of lysosomes follows a retrograde pathway, and the final destination of the lysosome depends on acidity and function. A recent study supports that spatial regulation of lysosomes is influenced by complete assembly of the lysosomal vacuolar (H^+^)-ATPase (V-ATPase) complex, with fully assembled V-ATPase-containing vesicles exhibiting mostly retrograde transport from the axon toward the soma (Verma et al., 2025). Perturbation of this transport process, such as disrupting microtubule stability, causes lysosomes to cluster near aggregated materials, potentially preventing distal degradation and increasing accumulation of autophagic vesicles (Zaarur et al., 2014).

Tau, a microtubule-associated protein abundant along axon microtubules, plays a vital role in maintaining cytoskeletal stability (Barbier et al., 2019). When hyperphosphorylated, tau detaches from microtubules (Lo, 2022; Lo and Sachs, 2020), leading to cytoskeletal disorganization and impaired cargo transport (Cowan et al., 2010). Similarly, genetic mutations of tau, such as tauP301L or tauP301S, have been shown to disrupt microtubule stability in vitro, which can impair vesicle transport (Combs and Gamblin, 2012). This microtubule instability has significant effects on lysosome mobility and therefore affects overall degradation capacity within the cell. In post-mortem brain samples from tauopathy patients, cathepsin D-spilling lysosomes accumulate around the nucleus with autophagic vesicles accumulating at distant locations from the soma (Piras et al., 2016). This relationship indicates a breakdown in lysosomal maturity, potentially due to an inability to transport these lysosomes from distal areas of the neuron, exacerbating the degradation issue of these toxic tau proteins.

Pathological tau not only obstructs microtubule tracks but also perturbs motor interactions, disrupting kinesin function (LaPointe et al., 2009). Interestingly, under normal conditions, patches of non-pathological tau regulate dynein and kinesin proteins, reversing the direction of dynein while preferentially influencing kinesin detachment (Dixit et al., 2008). This mechanism ensures balanced anterograde and retrograde transport. However, this suggests that when tau becomes hyperphosphorylated and detaches from microtubules, this regulatory pattern becomes dysregulated and leads to inefficient transport. Loss of tau from axons increases kinesin access to microtubules which may allow for more anterograde transport, but the overall effect is the disruption of cargo delivery and neuronal homeostasis.

Alpha-synuclein (αSyn), an IDP implicated in PD, interacts directly with lysosomal membranes. αSyn has been shown to bind LAMP2A to be taken into the lysosome for degradation (Cuervo and Dice, 1996). In dopaminergic neurons, αSyn overexpression leads to accumulation of acidic lysosomes in the soma and a slowing of retrograde transport (Lin et al., 2023), preventing degradation near the distal axon and potentially contributing to synaptic dysfunction (Eisbach and Outeiro, 2013). Interestingly, stimulation of the trafficking-related, small GTPase Rab7 in PD models has been shown to reduce αSyn toxicity (Szegö et al., 2022). In PD, it has been shown that lysosome, hydrolase, and substrate trafficking are disrupted by αSyn aggregates, worsening the disease pathology (Mazzulli et al., 2016). This suggests that lysosomal positioning and motility are tightly coupled to autophagosome-lysosome fusion and the efficient degradation of αSyn aggregates.

Recently, it was observed in a mouse model of AD that knocking down ATP citrate lyase (ACLY), an enzyme involved in the conversion of citrate into acetyl-CoA and oxaloacetate that is normally decreased in AD patients, destabilized microtubules, disrupted autophagic-lysosomal flux, and accelerated β-amyloid (Aβ) deposition (Lin et al., 2025). While this study highlights the relationship of Aβ, lysosomes, and microtubules, much less is known about how Aβ affects intracellular transport of lysosomes as compared to tau and αSyn. While Aβ impairs both the endolysosomal pathway and kinesins (Ari et al., 2014; Marshall et al., 2020), more research on the mechanisms of dysfunctional transport needs to be conducted to show intracellular lysosomal transport defects. In addition, the intracellular transport of lysosomes within astrocytes and microglia, under both basal and pathological conditions, remains poorly understood. As glial cells play essential roles in maintaining neuronal health throughout life, it is crucial to investigate their lysosomal trafficking mechanisms for understanding neurodegenerative disease progression.

## Intercellular lysosome transport

Among various mechanisms that enable intercellular movement and exchange of biological molecules (Goodenough and Paul, 2009; Ventela et al., 2006), the formation of TNTs appears to be the sole pathway that allows dynamic intercellular trafficking of organelles such as lysosomes. TNTs are F-actin structures that form cytoplasmic continuity between cells, promoting the exchange of organelles, proteins, and macromolecules (Onfelt et al., 2006; Gerdes et al., 2013). These cytoplasmic protrusions can span distances of approximately 10–250 μm and exhibit a wide range of diameters, normally between 50 and 700 nm wide (Zurzolo, 2021; Veranic et al., 2008; Drab et al., 2023). While most TNTs formed by following the characteristics of F-actin-based structures allowing for cytoplasmic continuity, TNT variations have been observed in vitro (Zurzolo, 2021). Some TNTs have been reported to contain microtubules (Yamashita et al., 2018), while some projections lack cytoplasmic continuity (Zurzolo, 2021). TNT structures have also been seen to terminate near neighboring cell membranes, or invaginate into the neighboring membrane without fusing (Chang et al., 2022). In a recent study, TNT-like non-synaptic filopodia were observed in *ex vivo* mouse somatosensory cortex slices, being the first examples of TNT-like structures observed in mature mouse brains (Chang et al., 2025).

Multiple drivers and pathways have been implicated in TNT regulation. Small GTPases, like CDC42, and the cytosolic protein tumor necrosis factor (TNF) alpha-induced protein 2 (TNFAIP2, also known as m-Sec) are key drivers that promote actin polymerization and membrane protrusion, aiding in the initiation of TNT formation (Hase et al., 2009). Their activity can be triggered by the inflammatory signal TNF, which activates NF-κB (Lo, 2025), increasing TNFAIP2 expression and activating CDC42 (Jia et al., 2018; Wójciak-Stothard et al., 1998). TNFAIP2 is typically expressed in both the initiating and receiving cells to begin actin remodeling and membrane extension, with kinesins being essential to facilitating cargo movement along these cytoplasmic extensions (Dixit et al., 2008). The kinesins function in both intercellular and intracellular transport, although tauopathies and synucleinopathies have been shown to inhibit or disrupt kinesin activity (Prots et al., 2013; Falzone et al., 2010).

Other players have been implicated within this process such as myosin-X (Myo10), specifically in neuronal TNT formation (Gousset et al., 2013). Myo10 may play a role in inducing TNTs by transporting cargo to ends of filopodia that aid in actin polymerization, contributing to elongation of filopodia and conversion into TNTs (Gousset et al., 2013; Uhl et al., 2018). A mechanical theory of TNT formation has also been proposed, where cells that exhibit physical contact create cytoplasmic bridges as they begin to relocate. Membranes stay physically connected as the cells distant themselves, with TNTs being the remnants of that physical connectivity (Driscoll et al., 2022). Insulin receptor substrate (IRSp53) has also been linked to TNT initiation through recruitment of VASP, an actin polymerase, which promotes localized actin remodeling essential for nanotube formation (Tsai et al., 2022). Another form of induction is oxidative stress, such as H_2_O_2_ treatment, which induces TNT formation through activation of the PI3K/AKT/mTOR pathway, with inhibitors of these pathways showing reduced TNT formation (Lin et al., 2024). Collectively, TNT formation is driven by cytoskeletal remodeling through small GTPases, TNFAIP2/m-Sec, and stress or inflammation-activated signaling pathways that initiate protrusion and nanotube extension.

Due to their fragile and dynamic nature, examining TNTs and their associated intercellular trafficking can be challenging. Fluorescent microscopy is employed for live imaging of these TNTs, mainly using cell cultures and more recently in isolated brain tissue sections from transgenic AD mice (Chang et al., 2025). Cell cultures can be treated with stressors, such as IDP aggregates or H_2_O_2_, to induce TNT formation (Scheiblich et al., 2024; Baker et al., 2025). Immunohistochemistry of F-actin or plasmids that fluorescently tag F-actin can be used in live-cell or tissue imaging. Fixed cells or tissues can also be imaged using a probe that binds to F-actin, phalloidin, coupled with a fluorophore (Baker et al., 2025). Similar approaches can be used to track lysosomes, with use of lysosome trackers to label live lysosomes, or expression of lysosome membrane proteins, such as LAMP1/2A, with fluorescent protein tags. While these approaches may be successful in tagging and monitoring of lysosomal movement across TNTs, the challenges encountered are related to the preservation and identification of TNTs for subsequent characterizations (Baker et al., 2025). Additionally, presence of cytonemes, similar F-actin-containing cytoplasmic protrusions, complicate the identification of TNTs, as they also influence organelle positioning and there is currently no known marker to specifically stain TNTs. However, cytonemes are unable to transfer organelles and can contain signaling protein receptors, being involved more in cell signaling, unlike TNTs (Casas-Tintó and Portela, 2019; González-Méndez et al., 2019).

In neurons, imaging shows transfer of lysosomes with toxic species through TNTs. Neurons under stress with toxic IDP aggregates can export defective, less acidic lysosomes to other cells for support in degradation (Abounit et al., 2016a). This exchange may represent a support network, where neurons offload toxic species to glia and glia support neurons by donating functional lysosomes. Communication is bidirectional in neuron-glia interactions, with neuron-microglia and neuron-astrocyte relationships exhibiting bidirectional exchange of organelles and proteins (Chen and Cao, 2021; Chakraborty et al., 2023). This allows for both neurons and glia to donate and receive lysosomes, enhancing communication through reciprocal movement of organelles in neural systems. There was also evidence of transfer of toxic protein aggregates such as αSyn within individual astrocytes and microglia (Rostami et al., 2017; Scheiblich et al., 2021), although glia-glia interaction between cell types via TNTs remains to be investigated. Although TNT-mediated lysosome transfer may initially dampen stress, the transfer can also facilitate the spread of disease through prion-like pathologic proteins and accumulation of diseased lysosomes (Abounit et al., 2016a). In tauopathy models, tau fibrils have been shown to be transferred intercellularly through TNTs. As observed in embryonic rat neurons, when exchanged to recipient cells, tau cargo propagates disease through prion-like abilities that cause endogenous tau misfolding and seeding (Tardivel et al., 2016). αSyn shows similar capabilities, as αSyn fibrils can propagate through TNTs contained within the lysosome (Abounit et al., 2016a). Aβ is also transported across TNTs, from neurons to astrocytes, causing propagation of toxic proteins, although it remains unclear whether they can be transported via lysosomes (Wang et al., 2010). These observations support the idea that TNTs exhibit both beneficial and detrimental effects with the trafficking of lysosomes. Selective export of lysosomes may serve as a short-term coping mechanism, but loss of control transforms it into an amplifier of disease spreading.

## Molecular determinants of lysosome transport selectivity

Lysosomes are not all equally mobile or transferable, as their fates appear to lie in the molecular compositions of their membranes. Variations in the ratios of LAMP1 and LAMP2A proteins that make up half of all lysosome membrane proteins, may signal distinct functions of the lysosome (Eskelinen, 2006). Gene ontology network analysis has shown that LAMP1 interacts more with transport proteins, while LAMP2A interacts more with synaptic proteins (Abouward et al., 2025). Composition of LAMP1/2A in the lysosomal membrane may influence intracellular (potentially LAMP1) vs. intercellular transport (potentially LAMP2A). Other membrane proteins, like VAMP7 or other SNARE proteins, may influence the fusion properties and destination of lysosomes. Lack of SNARE proteins provide a cluster of lysosomes that are unable to fuse with autophagosomes, potentially causing the lysosome to be exocytosed or transferred through TNTs (Tian et al., 2020). Small GTPases, such as Rab7, Rab9, and ARL8b, are also involved in the selectivity of this pathway and influence whether lysosomes engage in anterograde, retrograde, or intercellular movement (Kendrick and Christensen, 2022). The presence of these various players in the autophagy and lysosome transport pathways may determine the final destination and function of lysosomes.

The lipid composition of the lysosomal membrane provides an additional layer of selectivity. Phosphoinositides, particularly phosphatidylinositol 3,5-bisphosphate (PI(3,5)P_2_), regulate organelle trafficking by controlling Ca^2^^+^-dependent fusion events through activation of TRPML1 (Dong et al., 2010). Disturbances in PIKfyve, the kinase responsible for the phosphorylation to produce PI(3,5)P_2_, disrupts lipid composition balance, leading to swollen, immobile lysosomes commonly observed in lysosomal storage and neurodegenerative disorders (Cai et al., 2013). These highlight how lipids and membrane composition have the ability to influence lysosomal identity and trafficking. Furthermore, lysosomal acidity could serve as both a functional purpose and a transport signal. As lysosomes travel from the axon to the soma, they become fully acidified (pH 4.5-5), implying normal interactions with motor proteins (Ferguson, 2018a). Therefore, mechanisms of deacidification, such as inhibition of V-ATPase, may act as a regulator that determines lysosomal fate. This pH-dependent transport and localization could be the cause of selective lysosome export observed in stressed neurons.

## Multi-pathway regulation of lysosomal transport

We propose a two-step model where intracellular transport acts as a primary response and intercellular lysosome transport as a secondary attempt to prevent neuronal death, with both representing coordinated responses to facilitate cellular transport and mitigate cellular stress. Pathological stressors, such as IDPs, pro-inflammatory cytokines like TNF, and oxidative stress, disrupt lysosome function by interfering with acidification and V-ATPase assembly and activity (Kim et al., 2023; Wang et al., 2015; Colacurcio and Nixon, 2016; Kim et al., 2025). Reduced degradative capacity allows for accumulation of aggregates which can interfere with motor proteins, enhancing aggregation in the cell periphery (Durairajan et al., 2024; Lee et al., 2011). Furthermore, accumulation of toxic protein aggregates stresses the lysosome by damaging the membrane, weakening lysosomal integrity, and increasing proton leakage and deacidification (Rose et al., 2024). However, reacidification of impaired lysosomes has been shown to restore organelle function and improve the clearance of aggregates, easing lysosomal stress on cells (Arotcarena et al., 2022).

As a normal stress response, the transcription factor EB (TFEB) signaling pathway is activated to increase lysosome biogenesis and autophagic flux is upregulated to restore degradative capacity (Guerrero-Navarro et al., 2025). When cells experience lysosomal stress from accumulation of dysfunctional lysosomes or disrupted trafficking, TFEB upregulates coordinated lysosomal expression and regulation (CLEAR) genes to increase lysosome biogenesis and autophagy (Liu et al., 2021). This increased autophagy allows for the clearance of aggregates and may aid in lysosome transport restoration with a decrease in pathological stressors, like IDPs, that inhibit motor proteins, such as kinesins (LaPointe et al., 2009). Furthermore, lysosomal acidification influences transport as fully acidified lysosomes exhibit enhanced retrograde trafficking toward perinuclear regions, while less acidic vesicles are more prevalent in distal regions (Lie and Nixon, 2018). These links between stressors, acidification, aggregate degradation, and transport provide a foundation for understanding how lysosomal stress responses influence both intracellular positioning and intercellular exchange.

**Figure 1:**
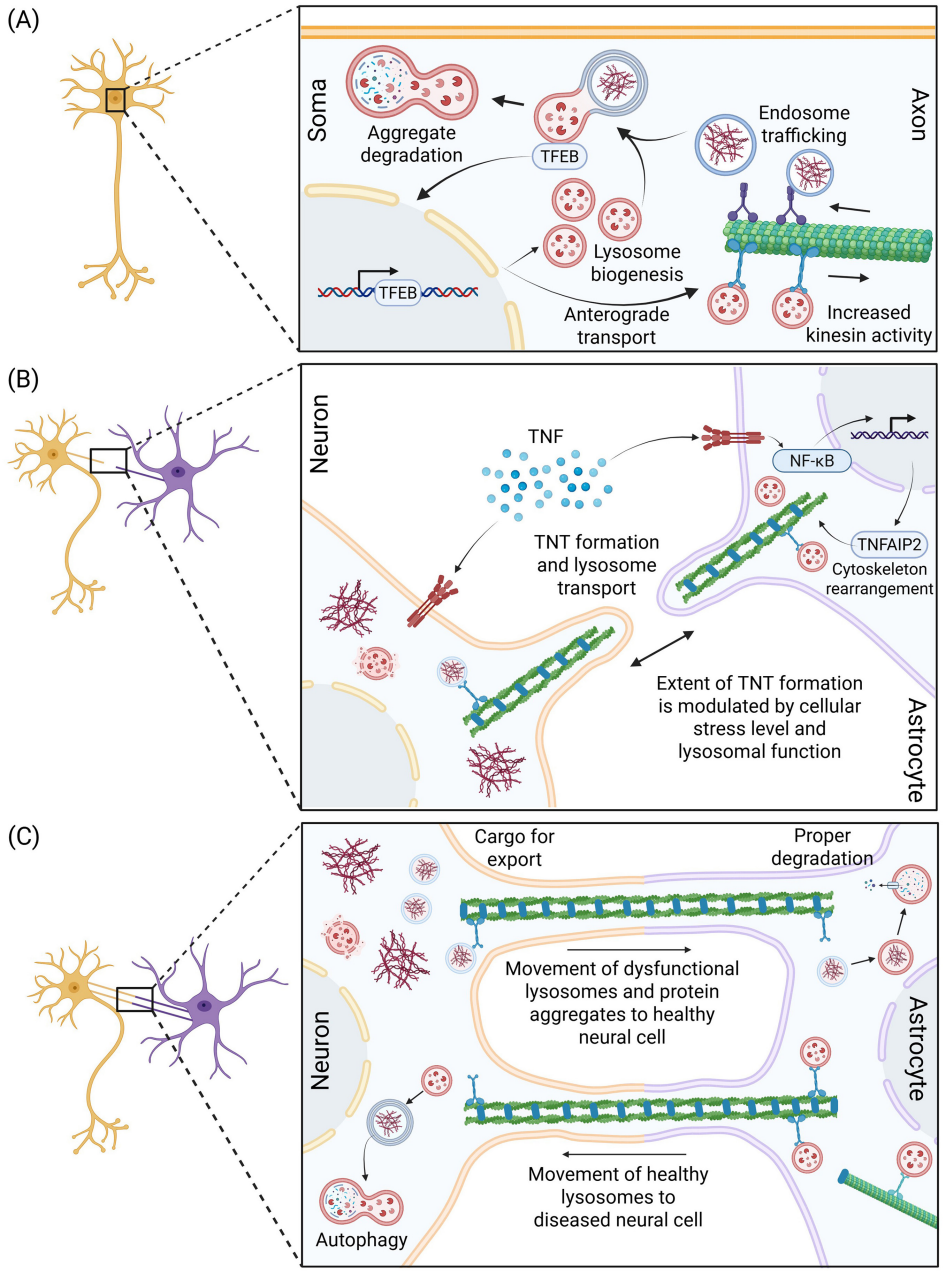
Schematic representation of multi-pathway regulation of lysosome transport. **(A)** Intracellular transport of aggregate-filled endosomes toward the soma allows for lysosomal fusion and degradation. Stress on the lysosome from aggregates causes TFEB to have downstream effects that turn on genes related to lysosome biogenesis and activates kinesins to increase anterograde transport of lysosomes to distal regions. Proper lysosome formation and transport allow for efficient aggregate degradation across the neuron. **(B)** Tunneling nanotube (TNT) formation induced by cellular stresses including inflammation, protein aggregation, and lysosomal dysfunction. Toxic aggregate stress causes TNF release, leading to activation of NF-κB and upregulation of TNFAIP2, an actin cytoskeleton regulator. TNT formation enables the exchange of lysosomes and aggregates between neural cells such as between neurons and astrocytes. **(C)** Selective lysosome transport between healthy and diseased neural cells playing a major role in maintaining cellular homeostasis. Diseased cells position toxic protein aggregates and defective lysosomes near the membrane for export and transport to healthy cells, where the protein contents can be properly degraded. Similarly, healthy cells transport functional lysosomes across TNTs to diseased cells which could assist in their cellular degradation.

Intracellular response: Under manageable stress conditions, neurons transport lysosomes within the cell to prevent a buildup of aggregates (Roney et al., 2022). Stress signals from aggregate buildups enhance ARL8-kinesin-BORC interactions for anterograde transport of lysosomes for aggregate degradation in distal regions (Wu et al., 2020). Motor adaptors enhance lysosome delivery near the nucleus, dendrites, and soma (Figure 1A). Intercellular response: When intracellular compensation fails, due to cytoskeletal disruption, acidification loss, or IDP accumulation, cells begin to form TNTs allowing for the exchange of IDPs and lysosomes (Abounit et al., 2016a). Toxic accumulation causes neural cells to become stressed, where TNF signaling and the NF-κB pathway are activated to induce TNT formation through cytoskeletal rearrangement in neurons and glial cells (Chen et al., 2024). Intensity of stress signaling, such as TNF level, may modulate the extent of TNT formation in cells (Wang et al., 2010; Matejka and Reindl, 2019). TNT formation facilitates healthy astrocytes for transportation of functional lysosomes to stressed neurons, while the neurons deposit defective ones (Chen and Cao, 2021). Neurons offload aggregate filled vesicles to astrocytes or other neurons to relieve stress for their own survival (Scheiblich et al., 2024). Selective lysosome movement across TNTs operates as an ordered cellular response that prioritizes neuronal survival by facilitating the bidirectional exchange of healthy and impaired lysosomes between neurons and astrocytes to preserve neuronal viability and utilize glia for aggregate clearance (Chakraborty et al., 2023; Figure 1B). As pathology progresses and overburdens both neuron and glia, this process breaks down, and the rescue response becomes a gateway for disease propagation (Abounit et al., 2016a,b). The same transport methods that once enabled rescue now serve as conduits for disease dissemination, moving non-functional, IDP-filled lysosomes that behave like seeds to propagate tau, Aβ, and αSyn pathology throughout neural circuits (Zhang et al., 2021).

Therapeutic exploration should assess whether promoting lysosome exchange or restoring lysosomal acidity can mitigate cellular stress. For example, promoting astrocyte-to-neuron lysosome transfer through TFEB (for increased lysosome biogenesis) and TNFAIP2 (for TNT formation) could determine whether replenishing acidic lysosomes restores neuronal function and reduces IDP accumulation (Xu et al., 2020; Ren et al., 2024; Fang et al., 2023). While there are currently no known molecules that exist to directly stabilize lysosome-motor protein interactions, trafficking can be stabilized through HDAC6 inhibitors and motor protein activators such as kinesore. HDAC6 inhibitors have been shown to stabilize microtubules, while kinesore is a molecule that activates kinesins, where synergistic treatment may allow for stabilized trafficking of lysosomes (Asthana et al., 2013; Randall et al., 2017). Small molecules (Vest et al., 2022; Chung et al., 2019) and acidic nanoparticles (Zeng et al., 2023; Lo et al., 2024) that enhance lysosomal acidification may represent tools to modulate lysosome positioning and reduce the intercellular spread of pathology. Together, these approaches will illustrate how selective lysosome trafficking across intracellular and intercellular systems establishes a dynamic network for maintaining proteostasis in the aging and diseased brain (Figure 1C).

## Conclusion

The lysosome is not only the endpoint of degradation but a dynamic organelle whose movement determines the survival or degeneration of neural cells. In neurons, selective lysosome transport within and between cells integrates degradation with cell-cell signaling and interactions. When properly regulated, this system allows precise spatial control of lysosomes and toxin mitigation under stressed conditions. When dysregulated, it becomes an unintentional vehicle for disease propagation. Hyperphosphorylated tau destabilizes microtubule networks and disrupts lysosomal transport. Aβ disturbs calcium homeostasis and lysosomal pH, while αSyn interrupts Rab-mediated trafficking and autophagic function. These disruptions produce an accumulation of defective lysosomes that are mispositioned, deacidified, and primed for spreading. Through TNTs, these defective lysosomes can expand to neighboring cells, propagating pathology and seeding disease across neural circuits. This suggests that neurodegeneration not only results from the accumulation of misfolded proteins, but from a breakdown in the spatial regulation of lysosomal transport. Disrupted trafficking, whether through impaired motor-adaptor affinity, loss of directional cues, or intercellular exchange of toxic species via TNTs, compromises the transport, delivery, and function of degradative organelles. TNTs also offer viable routes of healthy organelle transfer, promoting lysosome and mitochondrial transfer from healthy to pathologically burdened neurons or glial cells. These supplementary organelles may be able to promote survival through increased ATP production by mitochondria and decreased aggregate burdens through increased lysosome presence and activity (Scheiblich et al., 2024; Chakraborty et al., 2023). Therapeutic strategies that restore transport, stabilize lysosome-motor interactions, and regulate intercellular lysosome exchange may re-establish degradative capacity and slow the propagation of pathology across neural networks.
